# Knock-Out of IKKepsilon Ameliorates Atherosclerosis and Fatty Liver Disease by Alterations of Lipid Metabolism in the PCSK9 Model in Mice

**DOI:** 10.3390/ijms251910721

**Published:** 2024-10-05

**Authors:** Ulrike Weiss, Eleonora Mungo, Michelle Haß, Denis Benning, Robert Gurke, Lisa Hahnefeld, Erika Dorochow, Jessica Schlaudraff, Tobias Schmid, Silvia Kuntschar, Sofie Meyer, Rebekka Medert, Marc Freichel, Gerd Geisslinger, Ellen Niederberger

**Affiliations:** 1 Goethe University Frankfurt, Faculty of Medicine, Institute of Clinical Pharmacology, Theodor Stern Kai 7, 60590 Frankfurt am Main, Germany; weiss-ulrike@gmx.de (U.W.); mungo@med.uni-frankfurt.de (E.M.); hass@med.uni-frankfurt.de (M.H.); benning@med.uni-frankfurt.de (D.B.); gurke@med.uni-frankfurt.de (R.G.); hahnefeld@med.uni-frankfurt.de (L.H.); geisslinger@em.uni-frankfurt.de (G.G.); 2 Fraunhofer Institute for Translational Medicine and Pharmacology ITMP, and Fraunhofer Cluster of Excellence for Immune Mediated Diseases CIMD, Theodor Stern Kai 7, 60596 Frankfurt am Main, Germany; 3 Goethe University Frankfurt, Faculty of Medicine, Institute of Neuroanatomy, Theodor Stern Kai 7, 60590 Frankfurt am Main, Germany; schlaudraff@med.uni-frankfurt.de; 4 Goethe University Frankfurt, Faculty of Medicine, Institute of Biochemistry I, Theodor Stern Kai 7, 60590 Frankfurt am Main, Germany; t.schmid@biochem.uni-frankfurt.de (T.S.); silvia.kuntschar@gmail.com (S.K.); s.meyer@biochem.uni-frankfurt.de (S.M.); 5 Institute of Pharmacology, Ruprechts-Karl University Heidelberg, Im Neuenheimer Feld 366, 69120 Heidelberg, Germany; rebekka.medert@pharma.uni-heidelberg.de (R.M.); marc.freichel@pharma.uni-heidelberg.de (M.F.)

**Keywords:** atherosclerosis, fatty liver disease, PCSK9, IKKε, lipid, SCD1, FASN

## Abstract

The inhibitor-kappaB kinase epsilon (IKKε) represents a non-canonical IκB kinase that modulates NF-κB activity and interferon I responses. Inhibition of this pathway has been linked with atherosclerosis and metabolic dysfunction-associated steatotic liver disease (MASLD), yet the results are contradictory. In this study, we employed a combined model of hepatic PCSK9^D377Y^ overexpression and a high-fat diet for 16 weeks to induce atherosclerosis and liver steatosis. The development of atherosclerotic plaques, serum lipid concentrations, and lipid metabolism in the liver and adipose tissue were compared between wild-type and IKKε knock-out mice. The formation and progression of plaques were markedly reduced in IKKε knockout mice, accompanied by reduced serum cholesterol levels, fat deposition, and macrophage infiltration within the plaque. Additionally, the development of a fatty liver was diminished in these mice, which may be attributed to decreased levels of multiple lipid species, particularly monounsaturated fatty acids, triglycerides, and ceramides in the serum. The modulation of several proteins within the liver and adipose tissue suggests that de novo lipogenesis and the inflammatory response are suppressed as a consequence of IKKε inhibition. In conclusion, our data suggest that the knockout of IKKε is involved in mechanisms of both atherosclerosis and MASLD. Inhibition of this pathway may therefore represent a novel approach to the treatment of cardiovascular and metabolic diseases.

## 1. Introduction

Atherosclerotic cardiovascular disease represents the leading cause of mortality worldwide and is frequently associated with a number of comorbidities [1], including metabolic and obesity-related diseases, particularly metabolic dysfunction-associated steatotic liver disease (MASLD) and steatohepatitis (MASH) [2]. Atherosclerosis and MASLD are both multifactorial diseases that frequently manifest as a consequence of the metabolic syndrome, which is characterized by insulin resistance, dyslipidemia, and hypertension. These conditions often co-occur. In patients with MASLD, the most common cause of mortality is of cardiovascular origin (CVD) [3,4,5]. Furthermore, there is mounting evidence that MASLD is a risk factor for the development of atherosclerosis [5,6,7,8]. Nevertheless, the precise mechanisms underlying this correlation remain unclear. It was postulated that MASLD may contribute to the initiation of atherosclerosis by means of mixed lipidemia and hypercoagulable states. In particular, hyperlipidemia resulting in elevated concentrations of low-density lipoproteins (LDLs) in the blood, as well as the initiation of oxidative processes, are postulated to be key drivers of MASLD and atherosclerosis [9,10,11]. It is noteworthy that, while a number of treatment options exist for CVDs, there are currently only a few pharmacological options for the management of MASLD/MASH. Lifestyle modifications, including dietary changes and increased physical activity, represent the primary treatment strategies. However, long-term adherence to these recommendations is often challenging. Pharmacological agents targeting comorbidities such as type 2 diabetes, obesity, and dyslipidemia, including pioglitazone, GLP1 receptor agonists, SGLT2 inhibitors, and statins, are also recommended. Vitamin E is also discussed as a potential therapeutic option. However, these agents often exhibit only low efficacy. The thyroid hormone receptor agonist resmetirom demonstrated improvement in MASLD and MASH; however, the drug does not have worldwide approval, and the long-term evaluation of both desired and undesired effects is not yet available [12,13]. Therefore, the development of new and effective pharmacological interventions remains a crucial necessity. In addition to dysregulation of lipid metabolism, low-grade, non-resolving inflammation represents a significant contributing factor in the development of obesity, MASLD, and atherosclerosis [14,15]. The transcription factor Nuclear Factor kappa B (NF-κB) plays a pivotal role in both acute and chronic inflammation. It is activated in immune cells, as well as in liver cells and adipocytes, in the aforementioned conditions [16,17,18,19]. The non-canonical I-κB kinase epsilon (IKKε) is rapidly upregulated upon inflammatory stimulation [20,21]. It is involved in the activation of NF-κB by phosphorylation of IκBα, IKKβ, and the NF-κB subunits p65 or c-Rel [20,22,23,24,25,26]. Furthermore, it is a direct target of NF-κB signaling. Moreover, it plays a role in defense against viral infections through the phosphorylation of the interferon regulatory factors (IRFs) 3 and 7 [27,28]. IKKε, as well as its related kinase TBK1, have been previously associated with CVD and obesity [19,29]. Inhibition of IKKε by pharmacological treatment with the drug amlexanox or a knockout in mice resulted in enhanced insulin sensitivity, glucose tolerance, and improvement of fatty liver symptoms, which has been linked to elevated energy expenditure, diminished inflammation, and augmented catecholamine sensitivity [30,31,32]. The published data on the effect of IKKε inhibition on atherosclerosis are inconsistent. Some studies have shown no effect on atherosclerotic plaques, while others have indicated a reduction in the development of atherosclerosis. This discrepancy may be attributed to the use of disparate models, which vary significantly in terms of mouse genotypes, gender, dietary regimen, and the duration of the treatment [29,33,34].

In our study, we employed the proprotein convertase subtilisin/kexin type 9 (PCSK9) gain-of-function (GOF) model in conjunction with a high-fat diet [35,36,37], which has been demonstrated to induce atherosclerosis and fatty liver in mice without the necessity of genetic modification. This approach has the advantage of saving time and costs while also reducing cross-reactions, for example, in the case of different double knock-outs. The downregulation of LDL receptors by PCSK9 GOF results in elevated cholesterol levels in the blood [35,36] supported by a high-fat diet that induces mixed hyperlipidemia [38,39]. Experiments were initially performed in male mice only; however, in order to eliminate potential sex-specific differences, we repeated a number of experiments in a group of female mice. The findings indicate that the absence of IKKε impedes the onset and advancement of atherosclerosis and improves the prognosis of MASLD, however, particularly in male mice.

## 2. Results

### 2.1. Effects of IKKε Knock-Out in Mice on Weight Gain, Serum Lipid Levels, and Atherosclerotic Plaques

**Figure 1:**
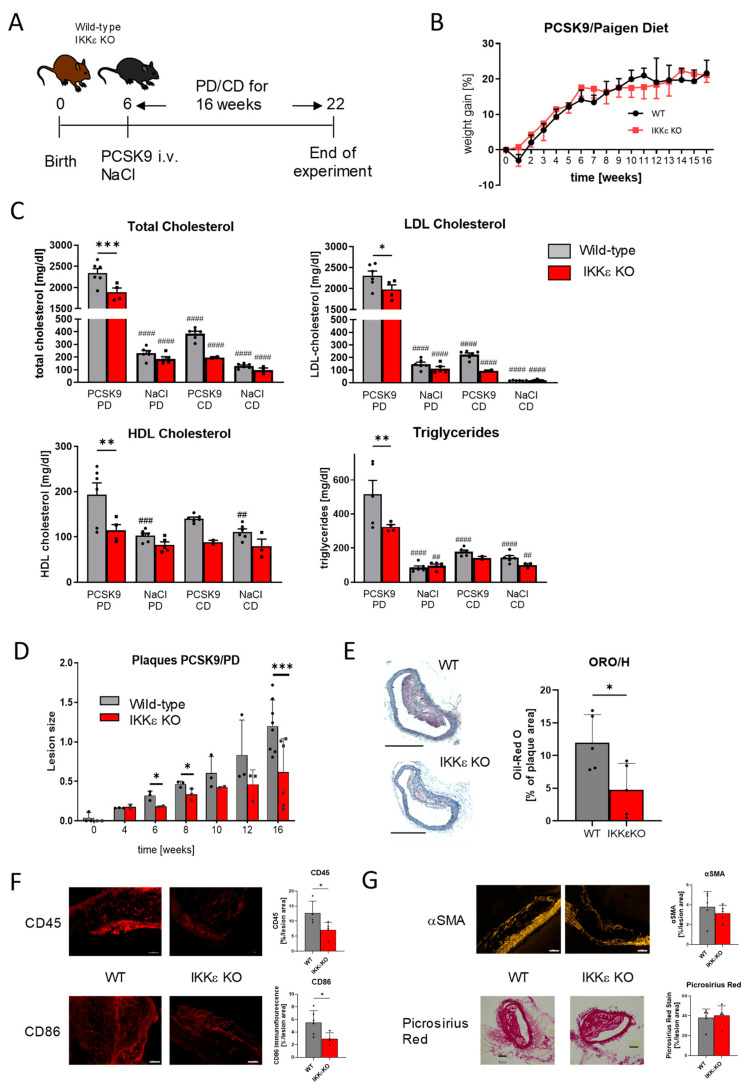
Weight gain, serum lipids, and plaque size in wild-type (WT) and IKKε knock-out mice. (**A**) Schematic overview of the treatment regimen of wild-type and IKKε knock-out mice. (**B**) Weight gain of male wild-type and IKKε knock-out mice after PCSK injection and Paigen diet (WT n = 5, IKKε KO n = 6), (**C**) serum lipid levels of wild-type and IKKε knock-out mice treated with PCSK9/PD, PCSK9/CD, NaCl/PD, and NaCl/CD (n = 2–6), one-way ANOVA, Tukey’s multiple comparisons test (**D**) time course of the lesion size of PCSK9/PD treated wild-type and IKKε knock-out mice (n = 2–6), Student´s *t*-test at each time point (**E**) plaque histology and quantitative analysis of the ORO/H stain (scale bar: 500 µm), (**F**) immunofluorescence staining and quantitative analysis for CD45 and CD86 in plaques of wild-type and IKKε knock-out mice (scale bar: 50 µm), (**G**) Picrosirius Red stain and αSMA immunofluorescence and quantitative analysis (scale bar: 100 µm), (**E**–**G**) representative pictures of at least three independent experiments, Student´s *t*-test. * *p* < 0.05, ** *p* < 0.01, *** *p* < 0.001 for comparison of genotypes, ## *p* < 0.01, ### *p* < 0.001, #### *p* < 0.0001 for comparison with control diet.

Male wild-type and IKKε knock-out mice were randomly assigned to receive either an intravenous injection of adeno-associated virus-8 (AAV8)-PCSK9^D377Y^ or 0.9% NaCl as a control, followed by a 16-week dietary intervention comprising a high-fat diet (Paigen Diet, PD) or a control diet (CD) (Figure 1A). Western Blot analysis confirmed the PCSK9^D377Y^ overexpression-induced decrease in LDL-receptor protein expression in the liver (Supplementary Figure S1). Mice that received the PCSK9^D377Y^ injections and were fed with PD gained approximately 20% of their initial body weight over the course of the treatment period, with no discernible difference between the genotypes (Figure 1B). At the end of the treatment period, total cholesterol (TC), LDL- and HDL-cholesterol, as well as triglyceride levels, exhibited a notable elevation in all mice within the PCSK9/PD cohort when compared to the NaCl and CD groups. These lipid regulations were all significantly alleviated in IKKε knock-out mice within the PCSK9/PD group. Furthermore, it has to be noted that cholesterol levels already showed a lower tendency in IKKε knock-out mice in the PCSK9/CD group (Figure 1C). A time-dependent analysis of atherosclerotic plaque development in wild-type and IKKε knock-out mice treated with PCSK9/PD revealed the presence of detectable plaques in wild type mice after a six-week treatment period, which exhibited a constant increase until the endpoint at 16 weeks. In contrast, the progression of plaques was delayed in IKKε knock-out mice, resulting in significantly smaller plaques (Figure 1D). Mice with NaCl and/or CD treatment developed no plaques. The quantification of histological staining with Oil Red O/haematoxylin (ORO/H) demonstrated a significantly reduced accumulation of lipid droplets in the plaques of IKKε knock-out mice (Figure 1E). Moreover, immunofluorescence analysis revealed a considerable presence of CD45-positive immune cells and CD86-positive pro-inflammatory macrophages within the plaques of wild-type mice, with markedly fewer of both in the IKKε knock-out mice (Figure 1F). Additionally, the potential impact on the uptake of oxLDL in primary macrophages of wild-type and IKKε knockout mice was evaluated but showed no discernible difference between the two genotypes (Supplementary Figure S2). Analysis of plaque stability via Picrosirius Red staining and immunofluorescence for smooth muscle actin (aSMA) in the plaque also revealed no differences between IKKε knock-out and wild-type mice (Figure 1G).

### 2.2. Effects of IKKε on Fatty Liver Disease

**Figure 2:**
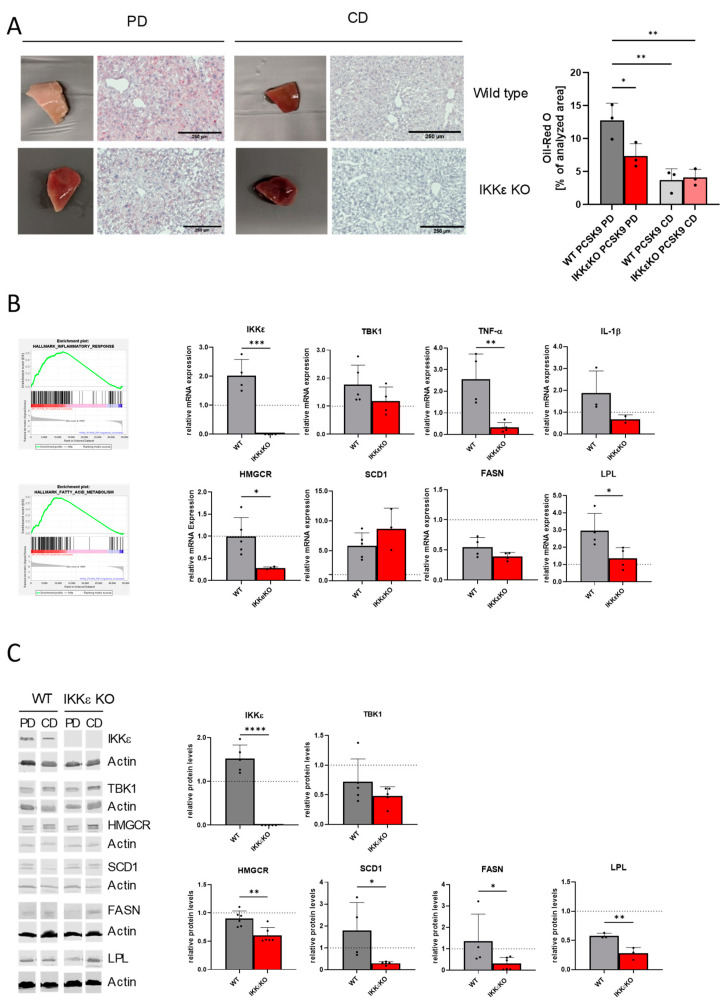
Effects of PCSK9/PD in livers of male wild-type and IKKε knock-out mice. (**A**) Liver pieces of wild-type and IKKε knockout mice fed with PD or CD. The histological stainings were performed with haematoxylin and Oil red O in liver slices of the respective mice. Quantitative analysis of (n = 3). One-way *ANOVA*, Tukey´s multiple comparisons test. (**B**) left side: GSEA analysis of RNA sequencing data of livers of male and female wild-type and IKKε knock-out mice treated with PCSK9/PD (n = 4–5). right side: mRNA regulations in the livers of male mice as assessed by RT-PCR analysis (n = 3–5). (**C**) Western Blot analysis of different proteins in the liver of male mice (n = 3–6). Protein bands were first normalized against the loading control β-actin and then against the respective control mice on CD. The dotted lines in (**B**,**C**) indicate the respective CD groups, which were set as 1 for better comparison. Student’s *t*-test. * *p* < 0.05, ** *p* < 0.01, *** *p* < 0.001, **** *p* < 0.0001.

Subsequent experiments were conducted to ascertain the influence of IKKε on the pathogenesis of fatty liver disease. In mice treated with PCSK9/PD, an increase in liver size and a pronounced accumulation of lipids were observed in comparison to mice treated with PCSK9/CD. The livers of IKKε knock-out mice exhibited a brownish coloration that was comparable to that of the CD-treated mice. Oil red O staining revealed fewer lipid droplets in liver slices of wild-type and IKKε knock-out mice on a CD diet than on a PD. The administration of PD resulted in a notable increase in lipid staining in wild-type mice, whereas this effect was not observed in IKKε knock-out mice, where diet-induced lipid depositions were found to be almost completely diminished. The quantification of ORO/H in the livers indicated that the observed differences were statistically significant (Figure 2A). The determination of serum glucose and liver glutamate oxaloacetate transaminase (GOT) revealed elevated levels of both markers in comparison to standard values (GOT ~48 U/L [40]; glucose ~136 mg/dL [41]) in wild-type mice treated with PCSK9/PD, indicating insulin resistance and disturbed liver function. The levels of glucose remained unaltered in the IKKε knock-out mice, while there was a tendency for the levels of liver enzymes to decrease (GOT WT: 185 ± 95 U/L, IKKε knock-out: 118 ± 3 U/L; glucose WT: 432 ± 49 mg/dL, IKKε knock-out: 408 ± 18 mg/dL) (n = 3–6). 

To identify potential mechanisms underlying the observed differences, we conducted an RNA sequencing analysis from liver tissue, which revealed, among other findings, an enrichment of genes involved in fatty acid metabolism and inflammatory responses in wild-type mice. These results align with the macroscopic and microscopic observations of liver alterations. Subsequent RT-PCR and Western blot experiments demonstrated an increase in IKKε expression in the livers of wild-type mice treated with PCSK9/PD. The levels of TBK1 were observed to be slightly and similarly elevated in wild-type and IKKε knockout mice that received PCSK9/PD treatment. TNF-α and IL-1β, which are indicative of liver inflammation, were elevated in comparison to CD-treated wild-type mice but exhibited a reduction in the absence of IKKε. In addition, we concentrated on the alterations in fatty acid and lipid metabolism and identified modifications in multiple genes. RNA and protein levels of HMG-CoA reductase (HMGCR), the rate-limiting enzyme in cholesterol synthesis, were significantly reduced in IKKε knock-out mice in comparison to wild-type mice. PCR analysis revealed no differences in Stearyl-CoA Desaturase-1 (SCD1), which is responsible for generating monounsaturated fatty acids (MUFAs), between wild-type and IKKε knock-out mice. However, diet-induced protein levels were significantly reduced in the liver of IKKε knockout mice. Moreover, we observed a reduction in the levels of lipoprotein lipase (LPL) and fatty acid synthases (FASN), which are involved in fatty acid metabolism and de novo lipogenesis (Figure 2B,C). 

**Figure 3:**
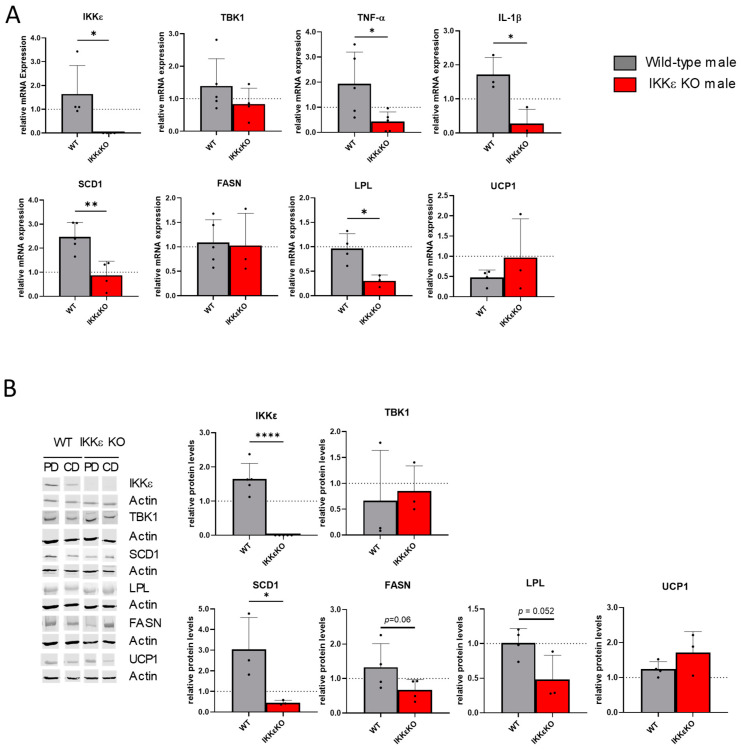
Gene regulations in adipose tissue of wild-type and IKKε knock-out mice. (**A**) mRNA regulations in WAT as assessed by RT-PCR analysis (n = 3–5). (**B**) Western Blot analysis of different proteins in the WAT (n = 3–6). Protein bands were first normalized against the loading control β-actin and then against the respected control mice on CD. The dotted lines in (**A**,**B**) indicate the respective CD groups, which were set as 1 for better comparison. Student’s *t*-test * *p* < 0.05, ** *p* < 0.01, **** *p* < 0.0001.

Given the established link between metabolic disorders and alterations in adipose tissue, we undertook a more detailed investigation into the transcriptional and translational changes occurring in white adipose tissue (WAT). In a manner analogous to that observed in liver tissue, IKKε in adipose tissue was slightly increased by PCSK9/PD treatment, while TBK1 remained unaltered. At the mRNA level, we also observed a reduction in inflammatory processes, as evidenced by the downregulation of TNF-α and IL-1β in WAT of IKKε knock-out mice. Similarly, SCD1, FASN, and LPL exhibited comparable regulatory patterns and were downregulated in IKKε knockout mice at the protein level. Since previous publications showed an increased energy metabolism in IKKε knock-out mice due to upregulations of UCP1, this protein was also investigated. The results showed a slight but not significant increase in UCP1 on mRNA and protein levels (Figure 3).

**Figure 4:**
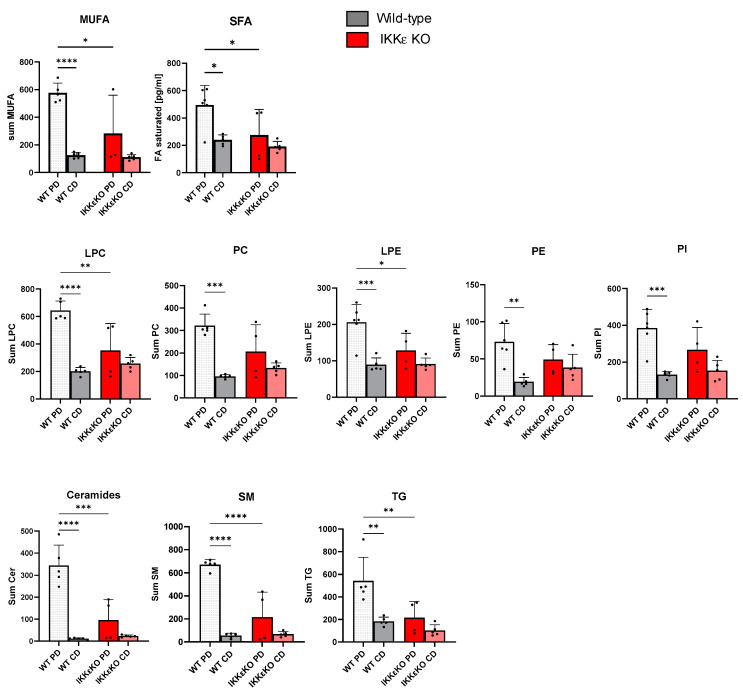
Lipid regulations in serum of wild-type and IKKε knockout mice. Lipid regulations in serum were determined by high-resolution mass spectrometry. For analysis, peak ratios relative to an internal for all lipids of specific lipid classes were summarized for wild-type and IKKε knock-out mice on PD or CD, respectively (n = 4–6). One-way *ANOVA* Tukey´s multiple comparisons test. **** *p* < 0.0001, *** *p* < 0.001, ** *p* < 0.01, * *p* < 0.05 (MUFA: monounsaturated fatty acids, SFA: saturated fatty acids, LPC: Lysophosphatidylcholine, PC: Phosphatidylcholin, LPE: Lysophosphatidylethanolamin, PE: phosphatidylethanolamine, PI: Phosphatidyinositol, Cer: Ceramides, SM: Sphingomyelins, TG: Triglycerides).

The observed regulations in metabolic genes in liver and adipose tissue may contribute to the modulation of lipid levels in IKKε knock-out mice, thereby improving MASLD. To evaluate the differential regulation of lipids in wild-type and IKKε knockout mice, we conducted lipidome analyses. The summation of lipids within the respective lipid classes demonstrated a significant increase in nearly all determined lipid groups in mice treated with the Paigen diet (Supplementary Figure S3). In IKKε knock-out mice on PD, there was a significant reduction in the levels of sphingomyelins, ceramides, LPE, and LPC, as well as triglycerides, in comparison to wild-type mice (Figure 4, Supplementary Figure S4). Moreover, a summation of all saturated (SFA) and all monounsaturated fatty acids (MUFAs) demonstrated a pronounced and statistically significant increase in wild-type mice following PD, which was markedly suppressed in IKKε knock-out mice (Figure 4).

### 2.3. Effects in Female Mice

**Figure 5:**
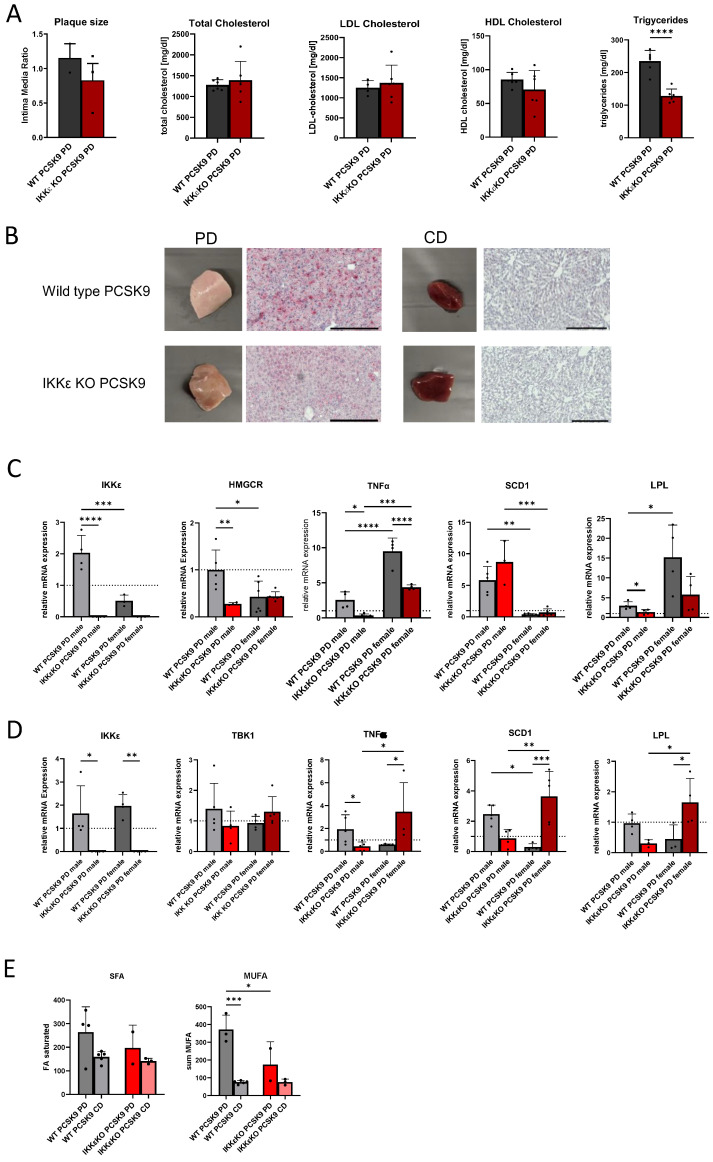
Effects of an IKKε deletion in female mice. (**A**) Lesion size of female PCSK9/PD treated wild-type and IKKε knock-out mice (n = 2–3) and serum cholesterol and triglyceride levels (n = 4–6), Students *t*-test **** *p* < 0.0001, (**B**) Liver pieces of female wild-type and IKKε knock-out mice fed with PD or CD. The histological stainings were performed with haematoxylin and Oil red O in liver slices of the respective mice. Representative picture of at least n = 3 (scale bar 200 µm). (**C**) Comparison of gene regulations in the liver of male and female mice (n = 3–6). (**D**) Comparison of gene regulations in the WAT of male and female mice (n = 3–6). The dotted lines in (**C**) and (**D** indicate the respective CD groups, which were set as 1 for better comparison. (**E**) Sum of saturated and monounsaturated fatty acids in the serum of male and female wild-type and IKKε knock-out mice (n = 2–5). One-way *ANOVA* Tukey´s multiple comparisons test. **** *p* < 0.0001, *** *p* < 0.001, ** *p* < 0.01, * *p* < 0.05.

The aforementioned results are based on experiments conducted on male mice. However, given the evidence from multiple studies indicating sex-based differences in the pathogenesis of fatty liver disease [42,43] and the observation by Patel that the inflammasome is more potently induced in female mice in an atherosclerosis model [33], we conducted additional experiments with a cohort of female mice. With regard to atherosclerosis, female IKKε knockout mice exhibited a tendency towards decreased plaque development, with no discernible impact on serum cholesterol levels. However, there was a notable reduction in serum triglycerides (Figure 5A). The appearance of the liver and the results of the ORO/H-staining were only slightly improved and were rather similar to those observed in wild-type mice (Figure 5B). This observation was corroborated by molecular analyses of genes in liver and adipose tissue. Following PD, the induction of IKKε in the liver was observed to be significantly lower in female mice compared to males. TNF-α levels were elevated in wild-type females relative to male mice; however, female IKKε knockout mice also exhibited a diminished inflammatory response. The analysis of metabolic genes revealed that the levels of HMGCR remained unaltered, which corresponded with the observed cholesterol levels in serum. Additionally, SCD1 remained unaltered while LPL exhibited an increase (Figure 5C). These regulatory processes point to marked differences compared to those observed in male mice. In adipose tissue, TNF-α, SCD-1, and LPL were increased in female IKKε knockout mice, representing a significant contrast to the regulations detected in male mice (Figure 5D). No change was observed in saturated fatty acids in IKKε knockout mice following PD, while monounsaturated fatty acids were decreased in comparison to female wild-type mice. Serum lipids were generally lower in female mice than in male mice but also demonstrated a significant increase following PCSK9/PD, which was ameliorated in IKKε knock-out mice (Figure 5E).

## 3. Discussion

The objective of the present study was to examine the influence of IKKε on the pathophysiology of atherosclerosis and liver steatosis in the PCSK9/PD model in mice. The results demonstrated that the absence of IKKε diminished the development and progression of atherosclerosis, as evidenced by a reduction in serum cholesterol and a decrease in immune cell activity. Furthermore, the deletion of IKKε was associated with decreased levels of several additional lipid species in the serum, which may contribute to improved fat deposition in the liver and the prevention of MASLD.

The role of IKKε in atherosclerosis and obesity has been previously investigated in other studies. However, the literature often presents conflicting findings. Cao et al. observed a reduction in weight gain and a decrease in the number of atherosclerotic plaques in ApoE/IKKε double knock-out mice as compared to ApoE single knock-out mice but no alterations in lipid levels. The authors proposed that the inhibition of IKKε-mediated NF-κB pathways may represent a potential underlying mechanism [34]. A study by Patel et al. employed the same model but observed an elevated inflammatory response in ApoE/IKKε knock-out mice, accompanied by augmented adiposity and inflammatory responses associated with enhanced inflammasome priming and activity, particularly in female mice. The size of the plaques in mice with a single or double knock-out was comparable; however, the double knockout mice exhibited smaller necrotic cores. Furthermore, the authors observed an increase in liver steatosis and cholesterol levels in ApoE/IKKε double knock-outs, accompanied by a reduction in Cyp7a levels in the liver [33]. These findings are inconsistent with those of a recent study that employed the IKKε/TBK1 inhibitor amlexanox. That study observed improvements in atherosclerosis and reductions of cholesterol levels due to increased bile acid excretion, which was mediated by an upregulation of Cyp7 levels in LDL-R knockout mice [29]. The partially contrasting results may be due to differences in the mouse genotypes, gender, diet, and duration of the treatment [29,33,34].

The present study was based on a model that has the advantage of not requiring multiple genetic modifications. PCSK9^D377Y^ overexpression and a high-fat diet were applied to wild-type and IKKε single knockout mice, resulting in a downregulation of LDL-R in the liver, which was associated with hyperlipidemia, atherosclerosis, and liver steatosis. Notably, the severity of these effects was significantly reduced in IKKε knockout mice, predominantly in males. Concerning the examination of aortic plaques, our data are in accordance with the findings of Zhao et al. and Cao et al. [29,34], which demonstrate that IKKε inhibition results in the reduction of plaque size. Additionally, the plaques of IKKε knockout mice exhibited reduced fat deposition and a decrease in immune cells, including macrophage levels. We postulated that alterations in the internalization of oxLDL might be associated with diminished inflammation. However, in vitro experiments utilizing primary macrophages did not substantiate this hypothesis. The stability of the plaques does not appear to be influenced by IKKε, as evidenced by the absence of alterations in collagen and smooth muscle actin. The decrease in atherosclerotic plaque size was accompanied by markedly diminished cholesterol levels in serum and reduced levels of HMG-CoA reductase in the liver of IKKε knock-out mice, the rate-limiting enzyme in cholesterol synthesis. A slight decrease in HMGCR in wild-type mice upon HFD may be attributed to a compensatory mechanism in response to dietary overload, as previously observed in other studies [44,45]. Furthermore, the reduction of HDL levels in IKKε knock-out mice has already been shown in earlier studies [29] and might be due to an increase in complete cholesterol catabolism [29] or differential HDL subtypes [46,47].

In addition to the alleviation of atherosclerosis, a significant improvement in liver steatosis was observed in male IKKε knockout mice. The development of a fatty liver is a consequence of an impaired balance between lipid uptake and synthesis, as well as fatty acid oxidation and export. An excess energy intake in the form of a high-fat diet results in the accumulation of free fatty acids (FFAs) in the liver due to the enhanced lipolysis in adipose tissue, de novo lipogenesis in hepatic cells, and the transport of dietary nutrients towards the liver. If the liver is chronically saturated, lipotoxicity arises, which is associated with mitochondrial dysfunction, an overproduction of ROS, and the formation of toxic lipid species, including lysophosphatidylcholines, diacylglycerols, and ceramides [48,49]. In particular, several ceramide species (e.g., Cer16:0, Cer18:0, and Cer24:1) and sphingomyelins have been previously associated with cardiovascular complications and endothelial dysfunction [50,51,52,53,54,55,56], as well as MASLD pathology [56]. In our study, lysophosphatidylcholines, diacylglycerols, and the aforementioned ceramide species demonstrated increased levels in wild-type mice following PD, whereas they exhibited a notable decline in the serum of IKKε knockout mice. These lipid regulatory processes may contribute to the prevention of steatosis [57] and additionally ameliorate mitochondrial ROS production and oxidative stress [58]. In addition to the observed alterations in lipotoxin regulations, we noted a significant reduction in the levels of saturated and monounsaturated fatty acids in IKKε-depleted mice, which suggests an impact on *de novo* lipogenesis. To further elucidate this hypothesis, we examined several enzymes in the liver and adipose tissue that play a role in fatty acid metabolism. The results showed several differences between wild-type and IKKε knock-out mice in the levels of LPL, SCD1, and FASN. Lipoprotein lipase (LPL) is expressed in adipose tissue but is typically absent in the adult liver [59]. However, it can be induced in the context of MASLD and MASH [60]. Elevated levels of LPL have been linked to an increase in triglycerides, which can ultimately result in the accumulation of lipids, lipotoxicity, and insulin resistance [61]. LPL has also been discussed as an important factor in atherosclerosis; however, reports are controversial and suggest both pro- and antiatherogenic effects [62]. FASN plays a role in promoting the synthesis of saturated fatty acids, particularly palmitate and stearate, in the liver and the storage of fat in adipose tissue. The resulting fatty acids serve as precursors for the formation of other FAs or complex lipids, including triglycerides and lipotoxins. SCD utilizes saturated fatty acids as a substrate for the biosynthesis of monounsaturated fatty acids, which are also essential precursors of triglycerides, phospholipids, and cholesterol esters. It is increasingly evident from a growing number of studies that aberrant expression and activity of SCD1 are associated with an elevated risk of a range of metabolic disorders, including obesity, MASLD, and type 2 diabetes mellitus [63,64,65]. Mice with reduced SCD1-levels exhibit enhanced fatty acid oxidation and diminished circulating lipids following high-fat feeding [66]. Accordingly, FASN and SCD1 inhibitors have been subjected to clinical trials with a view to ascertaining their potential utility in the treatment of patients with obesity or MASLD [67,68]. In conclusion, the reduced levels of LPL, FASN, and SCD1 in liver and adipose tissue of IKKε knockout mice are in accordance with the observed lower lipid accumulation in serum and reduction of liver steatosis. In addition to the effects on lipid metabolism, the IKKε knockout resulted in an alleviation of the inflammatory response in liver and adipose tissue, which is in line with the findings of previous studies [19,30,31].

Given the evidence from earlier reports indicating sex-specific differences in MASLD and atherosclerosis models [33,42,43], we conducted additional experiments using female mice. Notably, the mice exhibited diminished protection from atherosclerosis and a more severe form of liver steatosis than male mice, presumably due to several contrasting regulatory patterns in lipid metabolism and inflammatory responses. An increased malresponse has also been observed in the study conducted by Patel, who found elevated inflammasome activation in female mice [33]. These results suggest the existence of gender-specific differences and underscore the importance of analyzing both sexes, in particular when studying metabolic diseases.

In conclusion, our data demonstrate that IKKε inhibition hinders the onset and progression of atherosclerosis in mice by reducing immune cell invasion and fat deposition. Moreover, MASLD is alleviated by IKKε inhibition in male mice through the downregulation of SFAs, MUFAs, lipotoxins, and triglycerides in the serum, which is achieved by regulating enzymes involved in lipid metabolism. It is noteworthy that the results obtained from male mice did not exhibit a direct correlation with those observed in female mice, suggesting the presence of gender-specific differences.

## 4. Materials and Methods

### 4.1. Mice

Male and female wild-type C57BL/6J were obtained from Charles River Laboratories (Wilmington, MA, USA). Homozygous IKKε^−/−^-mice with a C57BL/6J background were purchased from The Jackson Laboratories, Sacramento, CA, USA (B6.Cg-Ikbketm1Tman/J). In these mice, the exons 4–6 of the IKKε gene were replaced by a PGK-neo cassette, resulting in an inactive protein. IKKε^−/−^ mice are viable, fertile, and healthy. Control genotyping was performed using the following primers as recommended by The Jackson Laboratories:
oIMR69165′-CTT GGG TGG AGA GGC TAT TC-3′Mutant ForwardoIMR69175′-AGG TGA GAT GAC AGG AGA TC-3′Mutant ReverseoIMR70485′-GGC CCA CCG AAG GGG ATG AAG G-3′Wild-type ForwardoIMR70495′-CTG CCC GCA AGC TGG ACG ATG AT-3′Wild-type Reverse

Mice were used for experiments at the age of 6–8 weeks; the number of male and female mice was matched between wild-type and IKKε knockout mice, and the ratio was almost equal for all experiments. Animals had free access to food and water and were maintained in climate- and light-controlled rooms (24 ± 0.5 °C, 12/12 h dark/light cycle). In all experiments, the European ethical guidelines for investigations in conscious animals were obeyed, and the procedures were approved by the local Ethics Committee for Animal Research (Regierungspräsidium Darmstadt FK/1004 and FK/1115). All efforts were made to minimize animal suffering and to reduce the number of animals used (in compliance with the ARRIVE and the Directive 2010/63/EU guidelines).

### 4.2. Induction of Atherosclerosis by PCSK9 GOF and High-Fat Diet

Adeno-associated viral vectors encoding the gain-of-function variant D377Y of the murine PCSK9 (rAAV8-PCSK9D377Y) under the control of liver-specific promoter were kindly provided by the Institute of Pharmacology, University of Heidelberg, Germany.. The viral particles were administered via the tail vein of mice at a dosage of 1.0 × 10^11^ viral genomes per mouse. Immediately following the injection, the mice were either placed on a high-cholesterol/high-fat Paigen diet (PD, containing 16% fat, 1.25% cholesterol, and 0.5% sodium cholate, Ssniff, Germany) to induce chronic hypercholesterolemia or were switched to an adjusted normal chow diet (CD, Ssniff, Germany). Additional control animals were i.v. injected with 0.9% NaCl and provided with either PD or CD. The diet was administered for a period of 16 weeks in the majority of mice. To investigate the time course of atherosclerosis initiation and progression, several groups of mice were treated for 4, 6, 8, 10, and 12 weeks. 

The body weight of the animals was measured at least once per week. During the initial four-week period of the study, a subset of mice in the PD groups (~4%) exhibited an inability to tolerate the provided food. The animals exhibited a pronounced reduction in body weight, which was identified as a termination criterion. As a result, the mice were excluded from further analysis. At the end of the treatment period, mice were sacrificed, blood was collected, and aorta, liver, and lipid tissue were excised. 

### 4.3. Analysis of Blood Samples 

Blood samples were obtained via cardiac puncture. The blood was allowed to clot for 10 min, after which serum samples were generated by separating the upper phase after centrifuging the blood at 2000× *g* for 20 minutes at room temperature without braking. The serum was rapidly frozen in liquid nitrogen and subsequently stored at −80 °C until further analysis.

The serum was analyzed for total cholesterol, LDL, HDL, and triglycerides, as well as glutamate oxaloacetate transaminase (GOT) and glucose levels. Therefore, the serum samples were diluted 1:3 with 0.9% NaCl. The analyses were conducted in the central laboratory of the University Clinic Frankfurt.

### 4.4. Analysis of Polar Metabolites and Lipids Using LC-HRMS

A detailed description of the methods can be found in a previous publication [69] and in Supplementary Methods. Briefly, 10 µL of serum was combined with 75 µL of internal standard solution in methanol (MeOH), 250 µL of methyl tert-butyl ether (MTBE), and 50 µL of 50 mM ammonium formate. The mixture was vortexed and centrifuged for 5 min at 4 °C. The upper phase was transferred to a vial for lipidomics analysis. The lower phase was re-extracted with 100 µL of a saturated MTBE solution (MTBE/MeOH/H_2_O, 10:3:2.5), vortexed, and centrifuged again for 5 minutes at 4 °C. The upper phase from this step was combined with the initial upper phase in the vial. The combined upper phases were evaporated under nitrogen at 45 °C and reconstituted in 100 µL of MeOH for lipidomic analysis. For the polar metabolite analysis, the lower phase was transferred to a glass vial, and any remaining upper phase residue was discarded, before evaporation under nitrogen at 45 °C. The dried residue was reconstituted in 100 µL of 50% acetonitrile. The study utilized a Vanquish Horizon UHPLC system paired with an Orbitrap Exploris 480 mass spectrometer (Thermo Fisher Scientific, Dreieich, Germany). Lipids were separated using a Zorbax RRHD Eclipse Plus C8 column (50 mm × 2.1 mm ID, 1.8 µm particle size) and a same-type pre-column (Agilent Technologies, Waldbronn, Germany). A 14-min binary gradient with water (0.1% FA, 10 mM ammonium formate) as eluent A and acetonitrile:isopropanol (2:3, *v*/*v*) with 0.1% FA as eluent B was employed. For polar metabolites, a SeQuant ZIC-HILIC column (100 mm × 2.1 mm ID, 3.5 µm particle size) with a same-type pre-column (Merck, Darmstadt, Germany) was utilized, and separation was achieved using a binary gradient of water (0.1% FA) as eluent A and acetonitrile (0.1% FA) as eluent B. System operation was managed via XCalibur software v4.4 and Tune Application 3.1 (Thermo Fisher Scientific, San Jose, CA, USA). Data analysis was conducted using TraceFinder software v5.1 (Thermo Fisher Scientific, San Jose, CA, USA). Identification of compounds was performed using the mzCloud offline library 2020 and LipidBlast VS68 libraries. A heated electrospray ionization (H-ESI) source facilitated MS data acquisition in full scan mode at a resolution of 120,000. MS2 spectra were obtained in a data-dependent manner (ddMS2) with a resolution of 15,000 and a cycle time of 600 ms.

Normalization of lipid results was performed using one internal standard per lipid class, while polar metabolite results were normalized with probabilistic quotient normalization. Due to insufficient sample material for a QC pool, system performance was verified using a mixture of human plasma QCs and reinjections of a single sample. Reporting molar concentrations based on the IS concentrations was omitted, given that the method employed (reversed-phase chromatography) enables robust relative quantification. However, it is important to note that the reported values should not be regarded as absolutely quantitative (µmol/mL). To provide a visual representation of this data, the peak area ratios are presented.

### 4.5. Histology 

The aortic arches, including brachiocephalic arteries, as well as parts of the livers, were prepared, fixated for 48 h in 2% PFA, and then embedded in O.C.T. compound (Sakura Finetek Europe B.V., Alphen aan den Rijn, Netherlands). These samples were sectioned at 8 and 10 μm thickness in a cryotome (Leica CM3050S), respectively. 

Frozen sections of aorta and liver were subjected to Oil-Red O/haematoxylin (ORO/H), aortas additionally to Picrosirius Red staining. For ORO/H staining, sections were fixed 5 min in 4% PFA, washed 5 min in isopropanol (60%), and then stained for 10 min (ORO solution: 0.6 g in 120 ml isopropanol). After four washes in isopropanol and water, sections were stained with haematoxylin for 6 min and then again washed with tap water. Afterwards, they were mounted with Aqua-Poly/Mount (Polysciences Europe, Hirschberg, Germany). For the collagen staining, slides were incubated with Picrosirius Red solution (Sigma-Aldrich, Deisenhofen, Germany) for 1 h at room temperature and then washed twice in acidified water (5 mL glacial acetic acid to 1 L of water) and mounted.

For histological analysis, images were captured on a Keyence BZ-X810 fluorescence microscope, and ImageJ software 1.54f was used for analysis. The plaque size was calculated as a ratio of the intima area/media area. ORO/H was quantified by the amount of red color in the tissues.

### 4.6. Immunofluorescence Staining

For immunofluorescence staining, frozen sections of aortas were washed twice with ice-cold PBS for 5 min and permeabilized with PBS containing 0.1% Triton X-100 for 10 min. Subsequently, the sections were blocked in 3% BSA/10% NGS in PBS for 1 h to reduce non-specific binding and then incubated overnight at 4 °C with primary antibodies against α-SMA (smooth muscle cell marker) (Invitrogen, Thermo Scientific, Darmstadt, Germany (PA5-85070), 1:200), CD45 (immune cell marker) (ebioscience, Thermo Scientific, Darmstadt, Germany (14-4801-82), 1:200), and CD 86 (proinflammatory macrophage marker) (Proteintech, Martinsried, Germany, (13395-1-AP), 1:200), dissolved in PBS/3% BSA. After rinsing in PBSTx (0.1% Triton), sections were incubated for 2 h at room temperature with Cy3-conjugated secondary antibodies (Sigma, Merck; Darmstadt, Germany, (C2306), 1:1.200) dissolved in PBSTx (0.1% Triton). Sections incubated without primary antibodies served as background controls. After final rinsing in PBS, the sections were coverslipped with Aqua-Poly/Mount (Polysciences Europe, Hirschberg, Germany). Images were captured using an inverted fluorescence microscope (Axio Observer.Z1, Zeiss, Germany) equipped with a monochrome CCD camera and ZEN3.0 software (Zeiss, Germany). The immunofluorescence images shown in the figures represent only a representative result obtained from at least three animals per group. Image analysis was performed with ImageJ 1.54f software. Therefore, images were converted to 8bit B/W. Then, a threshold was set and the fluorescence signals in the plaque area quantified.

### 4.7. Uptake of Ox-LDL in Primary Macrophages 

Bone-marrow-derived macrophages (BMDMs) were isolated from male or female C57BL/6J wild-type or homozygous IKKε^−/−^-mice. It was ensured that the mice of each experimental group were of the same gender and age. The mice were euthanized via CO_2_-inhalation and cardiac puncture, after which the femur and tibia were removed and cleaned of excess tissue. The bones were incised at the knee joint and placed in a punctured 500 µL-Eppendorf tube. Subsequently, the tube was transferred to a 1.5 mL Eppendorf Tube, which had been previously filled with 500 µL of medium. The tubes were subjected to centrifugation at 14,000 rpm for one minute at room temperature. The bone marrow was resuspended in RPMI medium, supplemented with FCS (10%), penicillin/streptomycin (100 U/mL), and macrophage colony stimulating factor (MSCF) (0.02 µg/mL), and cultured in a 12-well plate. Medium was changed one and three days after isolation. Incubation of BMDM started 7 days after their isolation. The medium was removed, and the cells were washed with prewarmed PBS. The cells were incubated with a solution of 25 µg/mL oxidized LDL (ox-LDL) complexed with the fluorescent-dye 1,1′-dioctadecyl-3,3,3′,3′-tetramethylindotricarbocyanine perchlorate (Dil) for 24 h. To quantify the uptake of the oxidized LDL, the fluorescent signal was measured. For this purpose, the medium was removed, the cells were washed with warmed PBS, and then incubated for 10 min at room temperature with 4% PFA. Subsequently, the cells were stained with DAPI, and the fluorescence was quantified. 

### 4.8. Western Blot Analysis

Liver and adipose tissue samples were homogenized in PhosphoSafe Extraction Buffer (Merck, Darmstadt, Germany) containing protease inhibitor (1 mM Pefabloc SC, Alexis Biochemicals, Lausen, Switzerland) using an Ultrathurrax instrument (T10 basic, VWR, Darmstadt, Germany). Samples were kept at room temperature for 3 min before the cell lysate was centrifuged at 14,000 rpm for 30 min at 4 °C in an Eppendorf centrifuge. The protein-containing supernatant was stored at −80 °C until further analysis. 

Proteins (30 µg) were separated electrophoretically by 10, 12, or 15% SDS-PAGE and then transferred onto nitrocellulose membranes by semidry-blotting (Bio-Rad, München, Germany). To control the quality of the transfer, all blots were stained with Ponceau red solution. Membranes were blocked for 60 min at room temperature in Odyssey blocking reagent (LI-COR Biosciences, Bad Homburg, Germany) diluted 1:2 in 0.1 M PBS, pH 7.4. Afterwards, the blots were incubated overnight at 4 °C with primary antibodies against IKKε (#3416), TBK1 (#3013), SCD1 (#2794), FASN (#3180), and UCP1 (#14670) (all 1:250, Cell Signaling Technology, Boston, MA, USA), LPL (1:500, Thermo Scientific, Darmstadt, Germany (MA535444)), LDLR (1:250, Abcam, Cambridge, Great Britain) (ab52818), and HMGCR (1:250, Invitrogen/Thermo, Darmstadt, Germany) (MA5/35242) in Odyssey blocking reagent diluted 1:2 in 0.1% Tween 20 in 0.1 M PBS. After washing three times with 0.1% Tween 20 in 0.1 M PBS, the Blots were incubated for 60 min with an IRDye 680-conjugated secondary antibody (LI-COR, Bad Homburg, Germany, (926-68071)), 1:10,000 in blocking buffer diluted 1:2 in 0.1% Tween 20 in 0.1 M PBS). After rinsing in 0.1% Tween 20 in 0.1 M PBS, protein-antibody complexes were detected with the Odyssey Infrared Imaging System (LI-COR Biosciences). β-actin (37 kDa) (1:1200, Sigma, Germany (#A5441)) was used as a loading control and detected with an IRDye 800-conjugated secondary antibody (LI-COR, Bad Homburg, Germany, (926-32210)), Densitometric analysis of the blots was performed with Image Studio Lite Software 5.2 (LI-COR, Biosciences).

### 4.9. Real-Time PCR

RNA was prepared from mouse livers and fat tissue using TRI reagent as described previously [26]. Two hundred nanograms of total RNA were used for the reverse transcription, which was performed with Random and Oligo-dT Primers (2:1 ratio) in a Verso cDNA Synthesis Kit (Thermo Scientific, Darmstadt, Germany). Twenty nanogram RNA equivalents were subjected to real-time PCR in a QuantStudio 5 Real-Time PCR system using the SYBR Select Master Mix (Rox) (Life Technologies, Austin, TX, USA). Expression of mRNA was assessed related to GAPDH mRNA. The following gene-specific primers were used:
IKKε FW 5′-GTACAAGGCCCGAAACAAGA-3′
RV 5′-TCCTCCACTGCGAATAGCTT-3′TBK1 FW 5′-TGCTTACCCCAGTTCTTGCA-3′
RV 5′-CCCCAGCACTTCTCCTGATC-3′TNF-α FW 5′-GCTGAGCTCAAACCCTGGTA-3′
RV 5′-CGGACTCCGCAAAGTCTAAG-3′ IL-1β FW 5′-GCAACTGTTCCTGAACTCAAC-3′
RV 5′-ATCTTTTGGGGTCCGTCAACT-3′HMGCR FW 5′-AGCTTGCCCGAATTGTATGTG-3′
RV 5′-TCTGTTGTGAACCATGTGACTTC-3′LPL FW 5′-TGTGTCTTCAGGGGTCCTTAG-3′
RV 5′-GGGAGTTTGGCTCCAGAGTTT-3′FASN FW 5′-CCCCAGCGGTAGAGAATAGC-3′
RV 5′-CTAGAGGGCTTGCACCAACA-3′ LDL-R FW 5′-CCTGATTGCTGCACCTCTCT-3′
RV 5′-TTCCCACCCACTCAAAGCAA-3′SCD1 FW 5′-CAAACACCCGGCTGTCAAAG-3′
RV 5′-CTCGGCTTTCAGGTCAGACA-3′UCP1 FW 5′-ATGGTTGGTTTCAAGGCCACA-3′
RV 5′-CGGTATCCAGAGGGAAAGTGAT-3′GAPDH FW 5′-CAA TGT GTC CGT CGT GGA TCT-3′
RV 5′-GTC CTC AGT GTA GCC CAA GAT G-3′

The cycle number at which the fluorescence signal crosses a defined threshold (Ct-value) is proportional to the number of RNA copies present at the start of the PCR. The threshold cycle number for the specific mRNA was standardized by subtracting the Ct-value of GAPDH from the Ct-value of gene-specific amplificates of the same sample, respectively. Relative quantitative level of samples was determined by standard 2^(ddCt)^ calculations and expressed as foldchange of a single reference control sample.

### 4.10. RNA-Seq of Liver Samples, Functional Annotation, and Pathway Analysis 

RNA was extracted from liver tissue of male and female mice using TRI Reagent as indicated above. The RNA concentration as well as the quality and integrity of RNA were controlled using RNA ScreenTape assays on a TapeStation 4150 (Agilent Technologies, Waldbronn, Germany) and Qubit RNA HS Assay Kits on a Qubit 3.0 Fluorometer (Thermo Fisher Scientific). Sequencing libraries were prepared according to the workflow of the Quant Seq 3′ mRNA-Seq V2 Library Prep Kit FWD with UDI (Lexogen, Vienna, Austria). During the process, an additional step was taken to calculate the optimal cycle number for the endpoint PCR. For this, the PCR Add-on Kit V2 for Illumina (M02096-2-0130) was used. The quality of cDNA libraries was assessed using HS-D1000 ScreenTape assays on a TapeStation 4150, and quantities were measured using Qubit dsDNA HS Assay Kits. Libraries were sequenced (single end, 75 cycles) using a P2 100-cycle kit on a NextSeq 2000 instrument (Illumina, San Diego, CA, USA).

To assign the samples to their ID, the client BaseSpace Sequence Hub from Illumina was used for demultiplexing. The data were analyzed using Lexogens own data analysis pipeline, now called Kangaroo, which was previously performed via the BlueBee genomics platform at the time the RNA-sequencing was performed. Gene set enrichment analysis (GSEA) was performed using the GSEA module [70] on the GenePattern platform [71].

### 4.11. Statistical Analysis 

Statistical analyses were performed with Graph Pad Prism (version 10; Graph Pad Software Inc., La Jolla, CA, USA). The significance level was set at *p* < 0.05 for all comparisons. Data are presented as mean ± standard deviation (SD). For all data, the D’Agostino-Pearson normality test was used. Comparisons of two groups were analyzed either with an unpaired Student’s *t*-test or a Mann–Whitney nonparametric test. Statistical analyses of more than two groups were performed with analysis of variance (ANOVA) and Tukey’s multiple comparisons test.

## Data Availability

The raw data supporting the conclusions of this article will be made available by the authors upon request.

## Supplementary Materials

The following supporting information can be downloaded at: https://www.mdpi.com/article/10.3390/ijms251910721/s1.
