# Supplementary material for: Knock-Out of IKKepsilon Ameliorates Atherosclerosis and Fatty Liver Disease by Alterations of Lipid Metabolism in the PCSK9 Model in Mice
Source: Int J Mol Sci. 2024 Oct 5;25(19):10721. doi: 10.3390/ijms251910721 (PMC11476531; doi:10.3390/ijms251910721)
Supplement: Supplementary file 1 [file ijms-25-10721-s001.zip › Suppl. Figures_IJMS_h.pptx]

## Slide 1
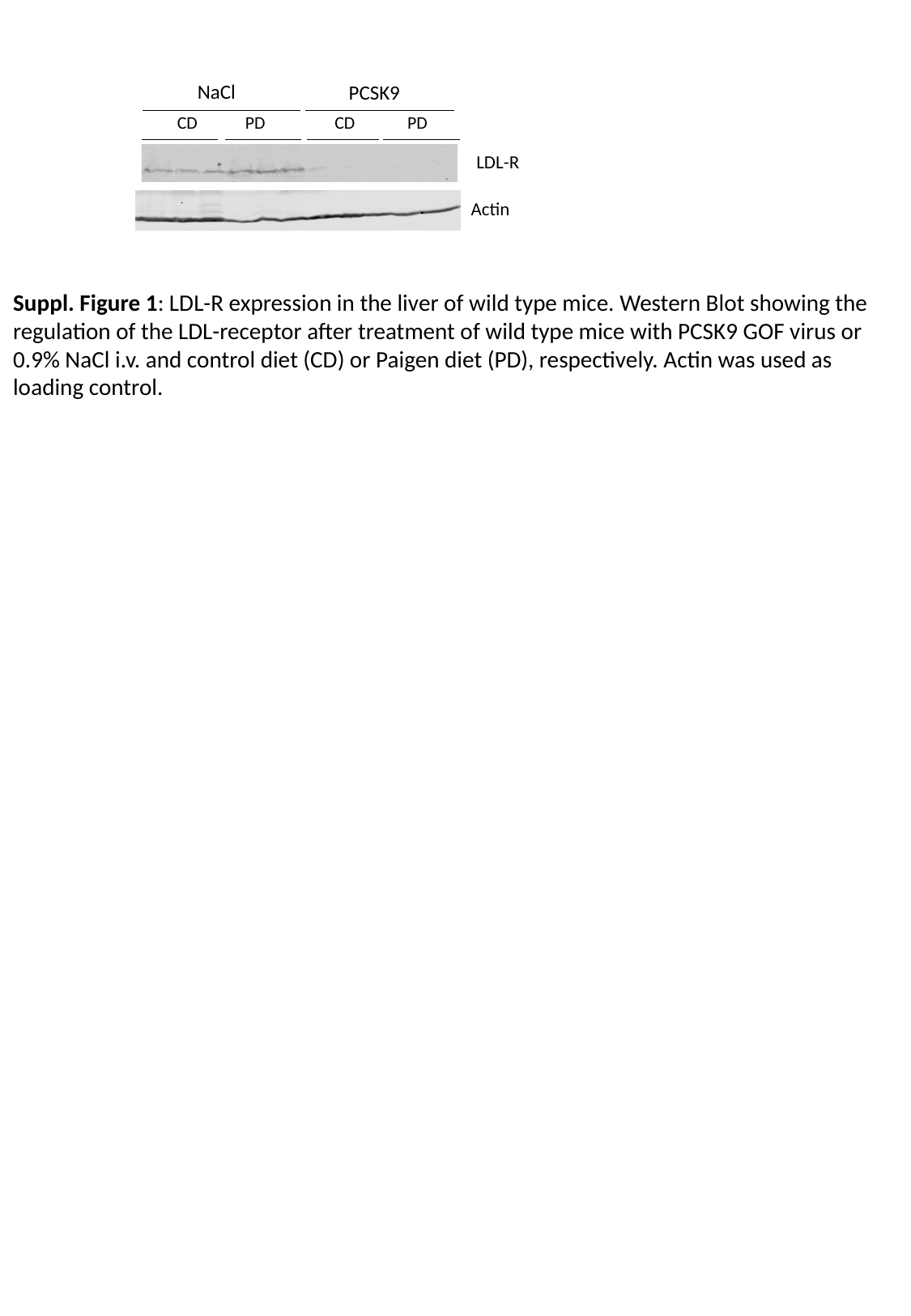

NaCl
PCSK9
CD
PD
CD
PD
LDL-R
Actin
Suppl. Figure 1: LDL-R expression in the liver of wild type mice. Western Blot showing the regulation of the LDL-receptor after treatment of wild type mice with PCSK9 GOF virus or 0.9% NaCl i.v. and control diet (CD) or Paigen diet (PD), respectively. Actin was used as loading control.

## Slide 2
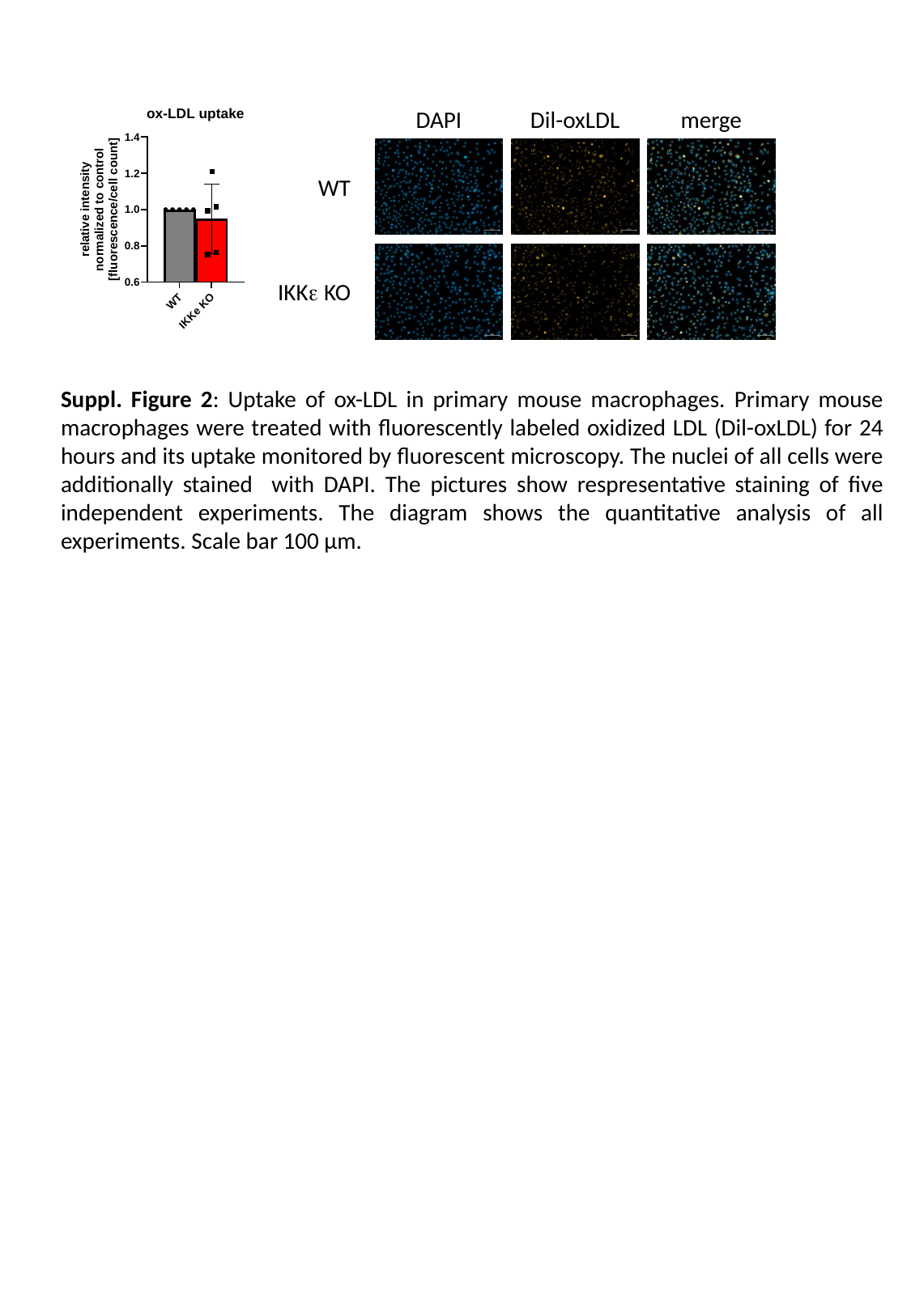

DAPI
Dil-oxLDL
merge
WT
IKKe KO
Suppl. Figure 2: Uptake of ox-LDL in primary mouse macrophages. Primary mouse macrophages were treated with fluorescently labeled oxidized LDL (Dil-oxLDL) for 24 hours and its uptake monitored by fluorescent microscopy. The nuclei of all cells were additionally stained with DAPI. The pictures show respresentative staining of five independent experiments. The diagram shows the quantitative analysis of all experiments. Scale bar 100 µm.

## Slide 3
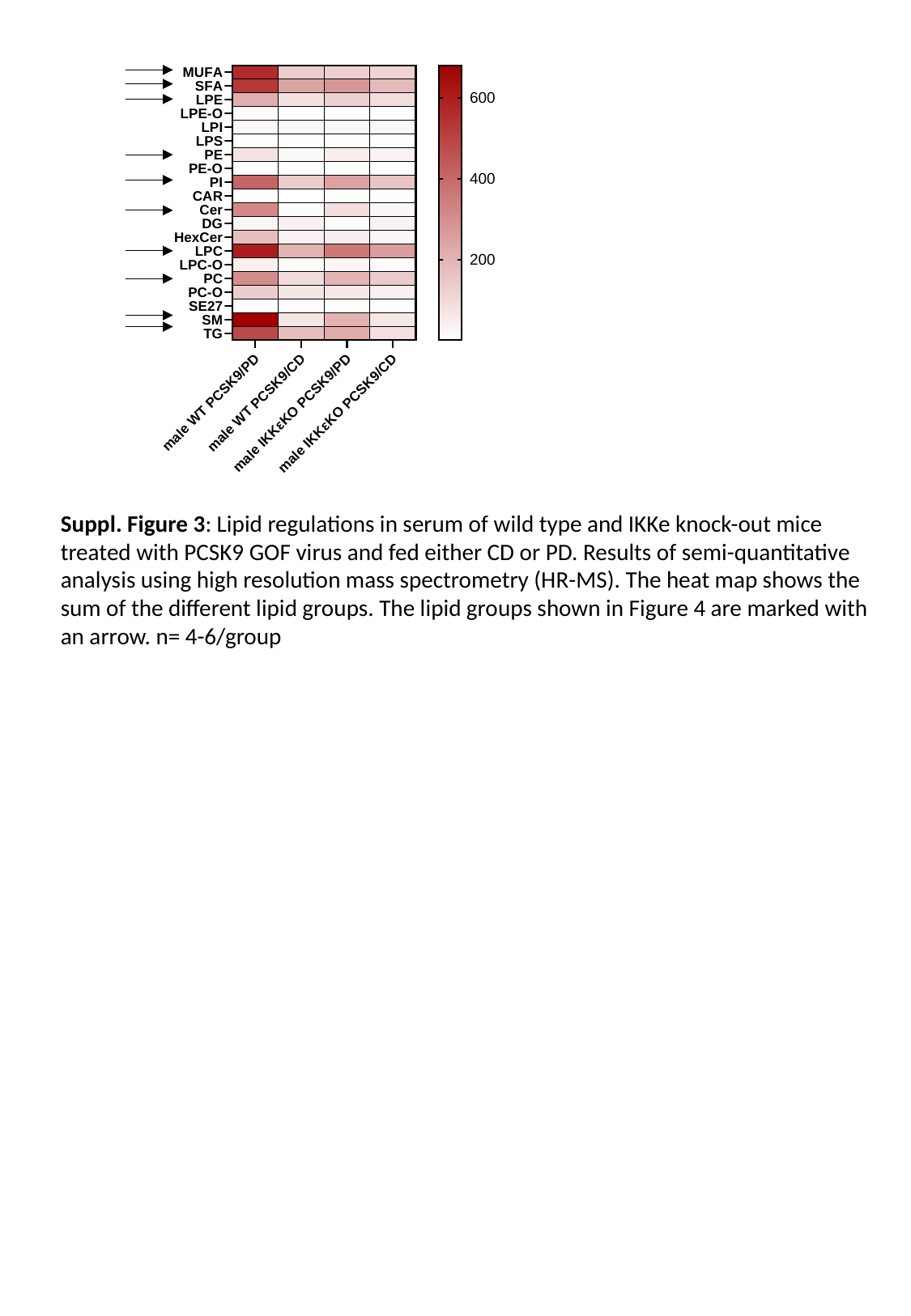

Suppl. Figure 3: Lipid regulations in serum of wild type and IKKe knock-out mice treated with PCSK9 GOF virus and fed either CD or PD. Results of semi-quantitative analysis using high resolution mass spectrometry (HR-MS). The heat map shows the sum of the different lipid groups. The lipid groups shown in Figure 4 are marked with an arrow. n= 4-6/group

## Slide 4
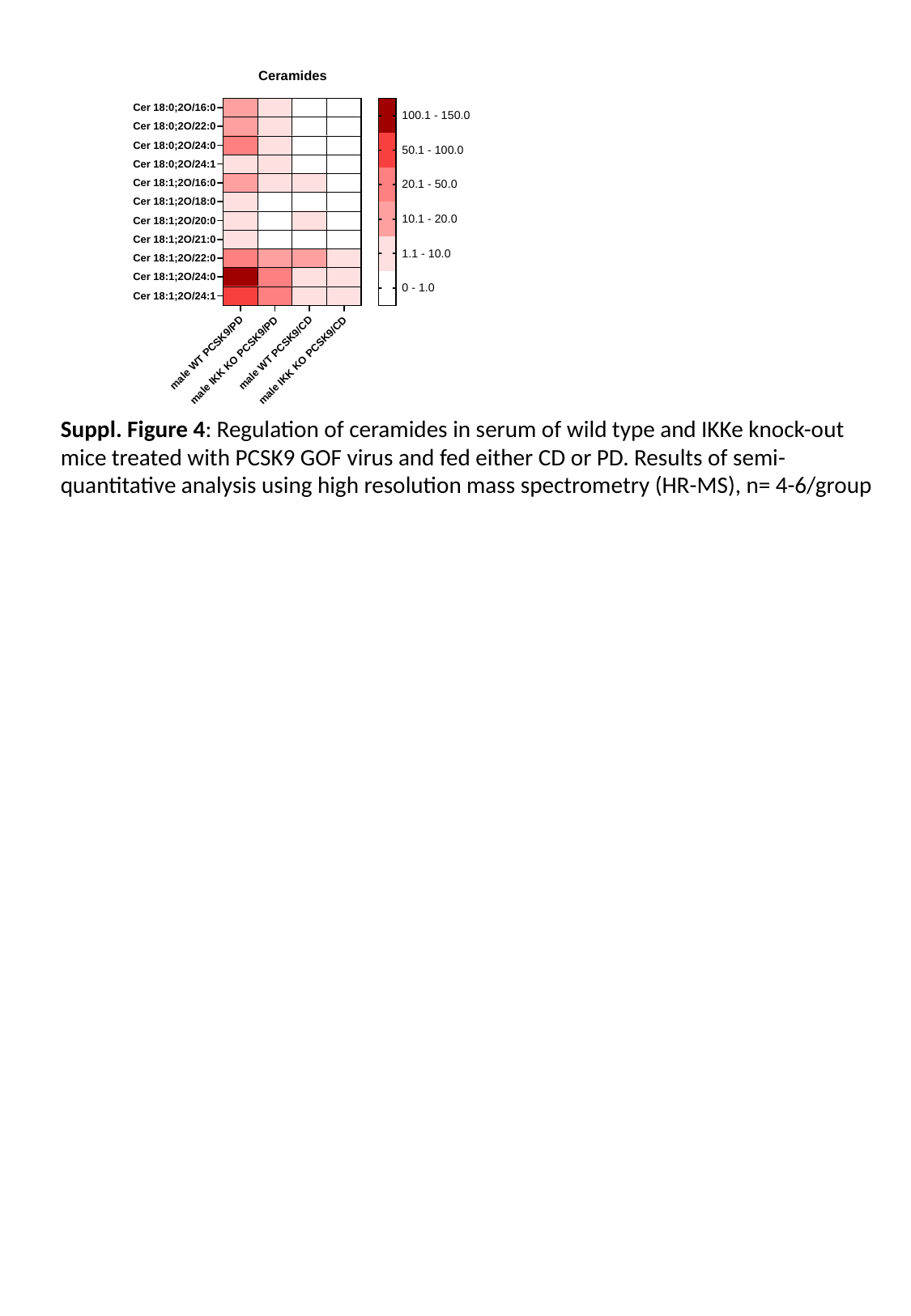

Suppl. Figure 4: Regulation of ceramides in serum of wild type and IKKe knock-out mice treated with PCSK9 GOF virus and fed either CD or PD. Results of semi-quantitative analysis using high resolution mass spectrometry (HR-MS), n= 4-6/group
